# Supplementary material for: Divergence in the Saccharomyces Species’ Heat Shock Response Is Indicative of Their Thermal Tolerance
Source: Genome Biol Evol. 2023 Nov 16;15(11):evad207. doi: 10.1093/gbe/evad207 (PMC10683043; doi:10.1093/gbe/evad207)
Supplement: evad207_Supplementary_Data [file evad207_supplementary_data.zip › SuppFigs.pdf]

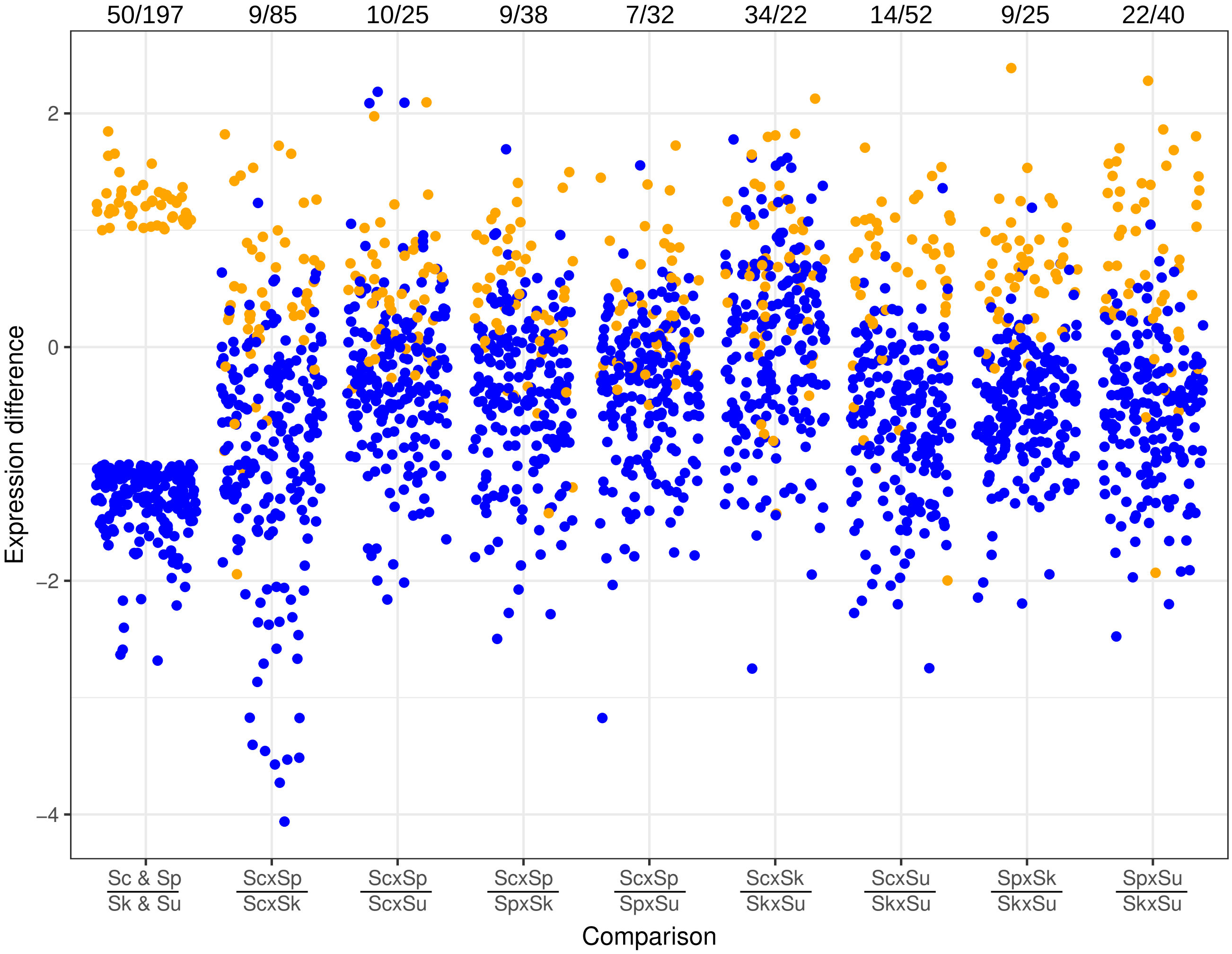

Figure S1. Hybrid expression differences in comparison to parental differences. Hybrid expression differences are shown in comparison to genes that differ two-fold between thermophilic (Sc & Sp) and cryophilic (Sk & Su) species. For the same genes, comparisons show differences between the ScxSp and other hybrids (ScxSk, ScxSu, SpxSk, SpxSu) and the SkxSu and other hybrids (ScxSk, ScxSu, SpxSk, SpxSu). Expression difference (log2) is the average response to heat across time-points and species' alleles for each comparison (delineated by horizontal line). Numbers at the top indicate the number of genes with differences greater than two-fold up/down..

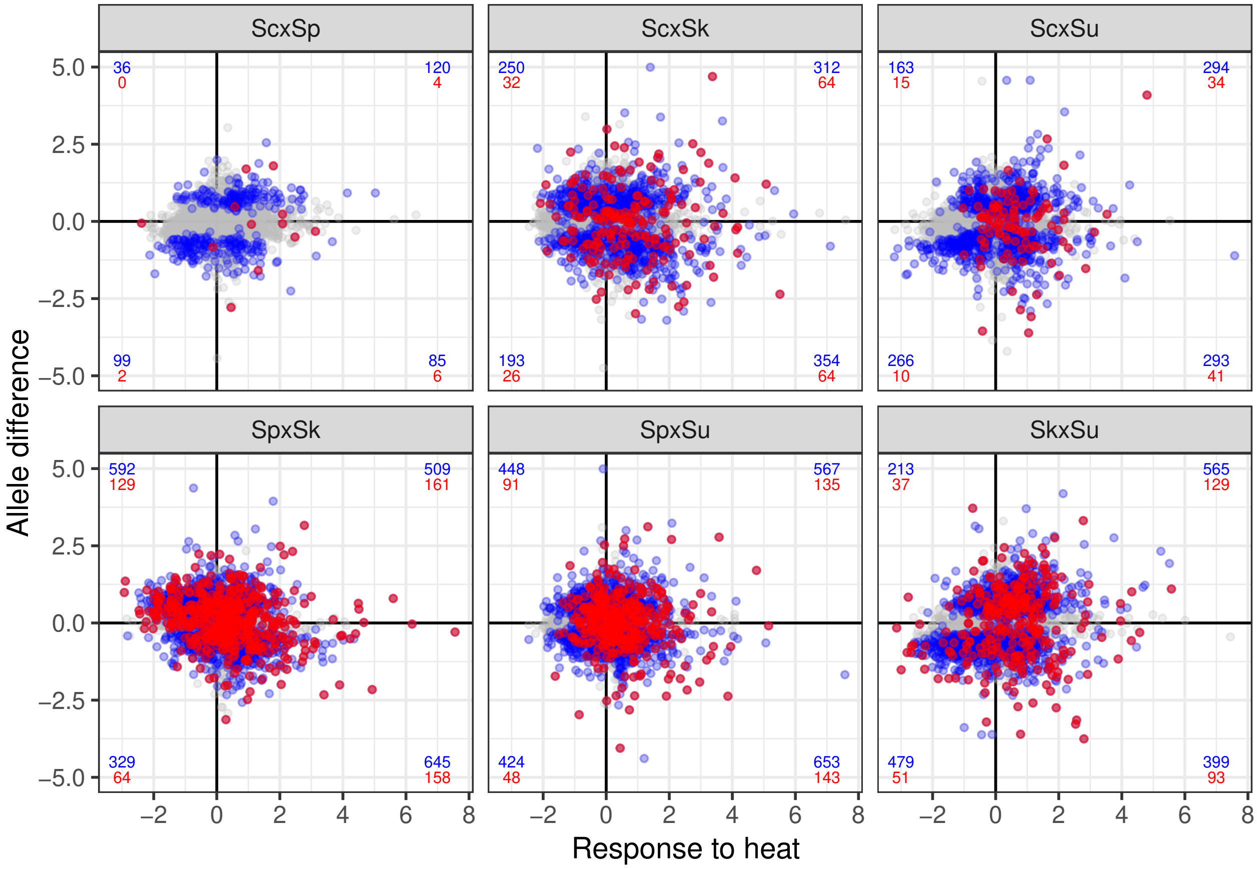

Figure S2. Relationship between allele differences and response to heat. The average allele difference across time-points (y, allele1/allele2 where alleles come from the hybrid name: allele1 x allele2) versus the average response to heat (x, normalized to the zero time point). Points show genes with significant allele and time effects (blue), genes with significant allele-time interactions (red), and all others (grey). Numbers in each quadrant indicate the number of genes with higher/lower expression for each species' allele and with an increase/decrease in response to heat, colored by significance category.

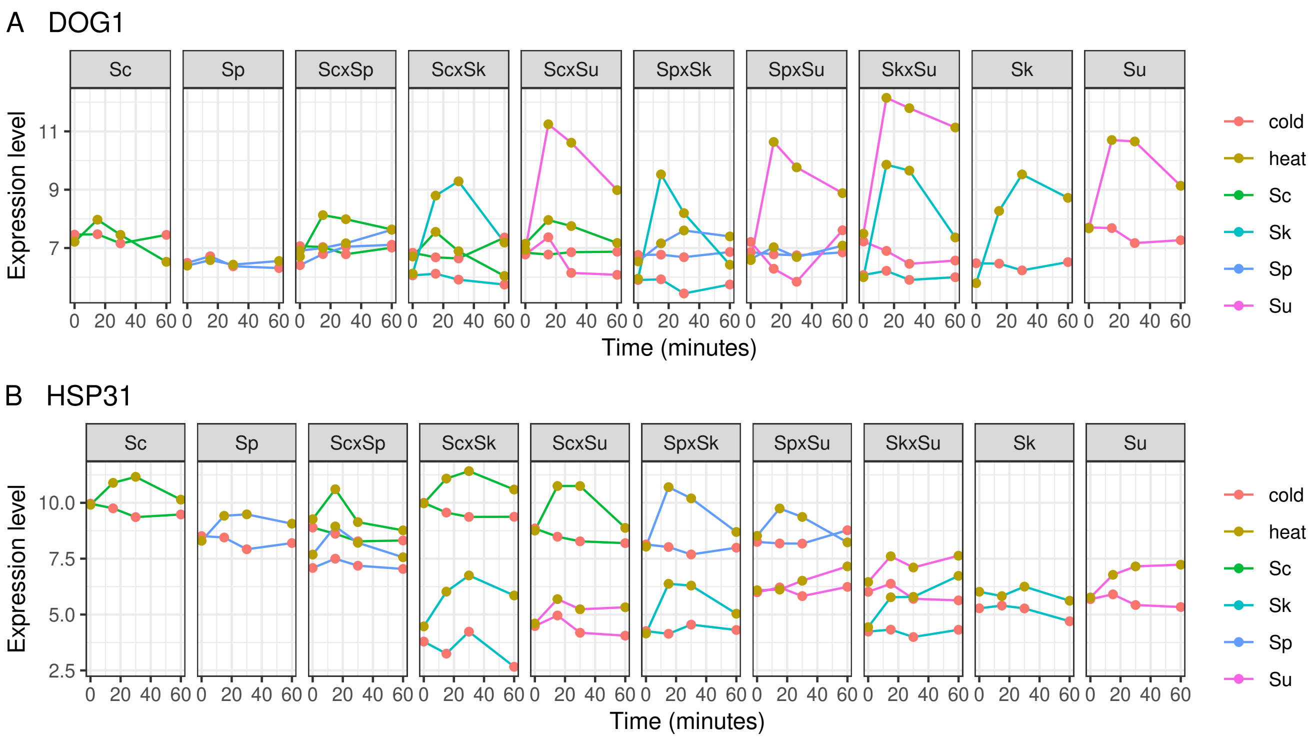

Figure S3. Expression of gene families in response to heat and cold treatment. For the *DOG1* family (A), *S. cerevisiae* and *S. paradoxus* expression is the sum of *DOG1* and *DOG2*, whereas *S. kudriavzevii* and *S. uvarum* expression is for a single copy. For *HSP31* (B) only *HSP31* expression is shown since one-to-one orthologs could be distinguished from other family members (*HSP32-34*).

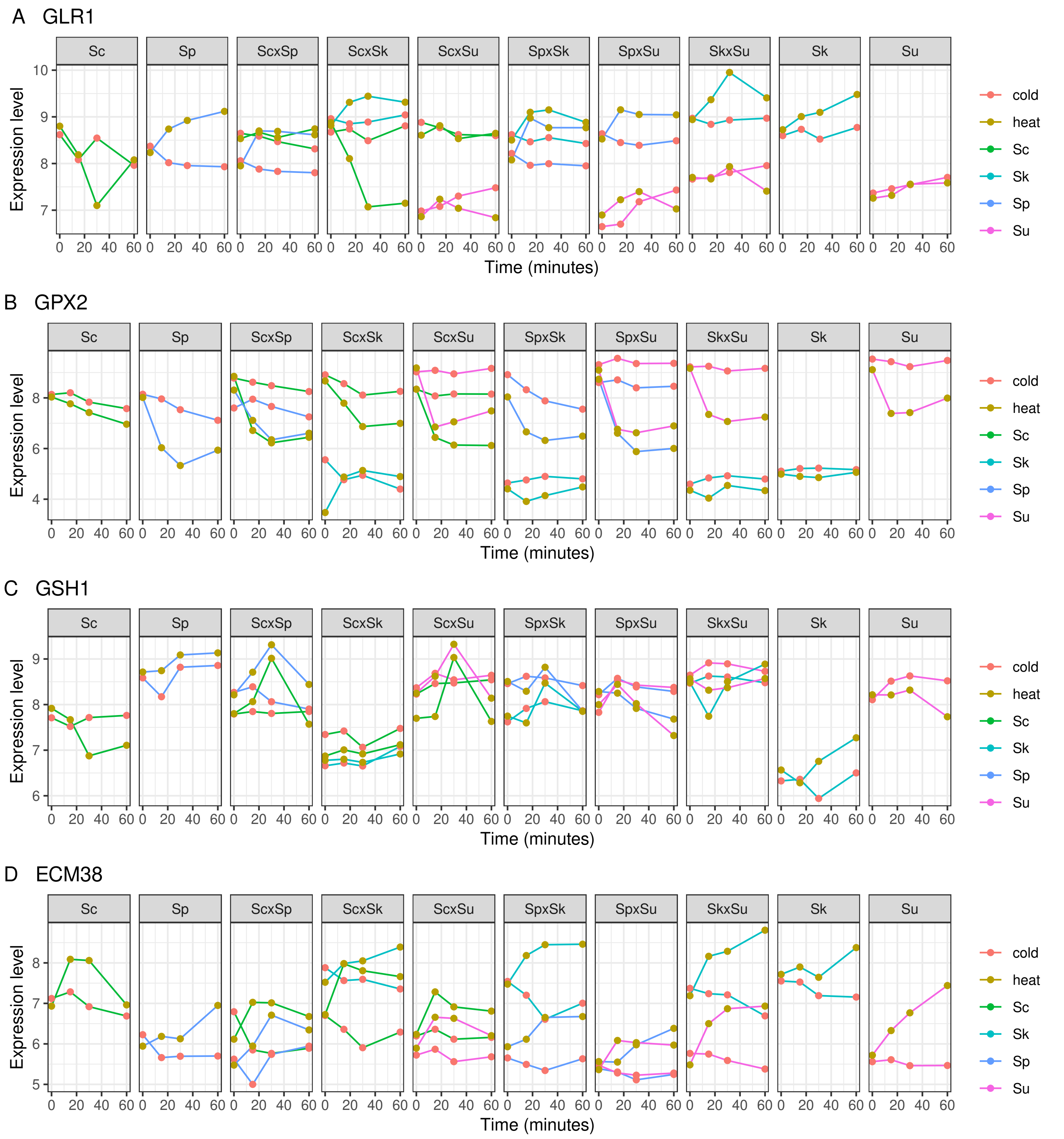

Figure S4. Expression of genes involved in glutathione metabolism: *GLR1* (A), *GPX2* (B), *GSH1* (C) and *ECM38* (D).
